# The Utility of IgG4/IgG Ratio in the Diagnosis of Multicentric Castleman Disease: A Case Report of HHV8+ Castleman Disease in a Patient with Classical Hodgkin’s Lymphoma

**DOI:** 10.3390/hematolrep18020026

**Published:** 2026-04-03

**Authors:** Adam Hagele, Philip Kay, Kevin Nishino, Akhil Mehta, Yan Liu, Anthony L. Nguyen, Eric Lau

**Affiliations:** 1 Department of Medicine, Loma Linda University Medical Center, Loma Linda, CA 92350, USA; 2 Independent Researcher, Orinda, CA 94563, USA; 3 Houston Methodist Dr. Mary and Ron Neal Cancer Center, Houston, TX 77030, USA; 4 Department of Pathology, Loma Linda University Medical Center, Loma Linda, CA 92350, USA; 5 Division of Hematology/Oncology, Department of Medicine, University of California San Diego, La Jolla, CA 92093, USA; 6 Department of Hematology and Oncology, Palo Alto Medical Foundation, Palo Alto, CA 94301, USA

**Keywords:** Multicentric Castleman Disease (MCD), HHV8-associated Castleman Disease, IgG4/IgG ratio, IgG4-related disease (IgG4-RD), fever of unknown origin (FUO), classical Hodgkin lymphoma, lymphoproliferative disorders diagnosis

## Abstract

**Background/Objectives**: Multicentric Castleman Disease (MCD) is a rare lymphoproliferative disorder that can mimic IgG4-related disease (IgG4-RD), particularly in patients presenting with elevated serum IgG4. Accurate diagnosis is crucial given differing treatments and prognoses. **Case Presentation:** We describe a 76-year-old male with fever, lymphadenopathy, and elevated inflammatory markers. Labs revealed an elevated IgG4 of 133 mg/dL and total IgG of 1410 mg/dL, yielding an IgG4/IgG ratio of 9.43%. Lymph node biopsy showed nodular sclerosing classical Hodgkin lymphoma, for which he received five cycles of A + AVD. Persistent symptoms, elevated IL-6, and HHV8 viremia prompted repeat biopsy, which demonstrated HHV8-positive MCD. Rituximab was initiated, which resulted in clinical and radiographic resolution. **Methods**: We performed a systematic review of the English-language literature from 2000 to 2025, identifying 23 studies that contained MCD cases with individual-level serum IgG4 and IgG data. A total of 36 unique cases were included. **Results**: The mean IgG4/IgG ratio was 14.61%, which is substantially lower than ratios typically seen in IgG4-RD. To our knowledge, our case is the only reported instance of HHV8-associated MCD with elevated IgG4. **Conclusions**: A mildly elevated IgG4/IgG ratio may favor the diagnosis of MCD over IgG4-RD. Serum IgG4 and total IgG should be considered when suspecting Castleman Disease.

## 1. Introduction

Multicentric Castleman Disease (MCD) is a rare lymphoproliferative disorder that often presents with fever of unknown origin (FUO) and elevated IgG4 levels. Elevated serum IgG4 levels have more commonly been attributed to IgG4-related disease. Serum IgG4/IgG ratio has been reported as a potential marker to distinguish between cases of MCD and IgG4-related disease (IgG4-RD), with the latter having markedly elevated ratios [1].

We report the case of a patient who presented with FUO, diffuse lymphadenopathy, modestly elevated serum IgG4/IgG levels and HHV8 viremia, and was found to have advanced nodular sclerosing Hodgkin’s lymphoma (NSHL). Although initially achieving a complete remission with Brentuximab Vedotin with Adriamycin, Vincristine, and Dacarbazine (A + AVD), recurrent lymphadenopathy at the end of treatment and persistent elevated serum IgG4/IgG levels and HHV8 viremia led to a biopsy-proven diagnosis of HHV-8-associated MCD. In addition, we report the results of a systematic literature review on serum IgG4/IgG levels of patients diagnosed with MCD in the English-language literature. Through our case presentation and literature review, we suggest that serum IgG4 and IgG levels may be helpful as part of the workup of a fever of unknown origin (FUO).

## 2. Case Description

**Figure 1:**
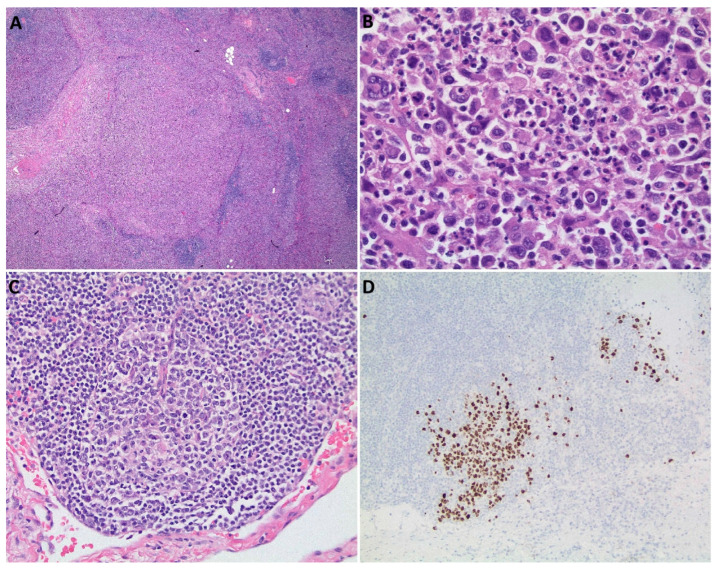
Pathology: Initial Hodgkin’s lymphoma diagnosis from inguinal lymph node biopsy: (**A**): H&E: Nodal architecture is effaced by multiple nodular lymphohistiocytic infiltrates surrounded by collagenous bands. (**B**): H&E: Multiple Reed Sternberg Cells against an inflammatory background in the nodules, which have membranous CD30 and CD15+, are weakly PAX5+, and are negative for LANA/HHV8 (not pictured). Subsequent HHV-8-associated Multicentric Castleman Disease diagnosis from cervical lymph node biopsy: (**C**): Lymphoid follicle with onion-skin-appearing mantle zones and penetrating hyaline vessel with classic lollipop appearance. (**D**): Immunohistochemistry stain positive for HHV-8 in variable numbers of the follicular center cells in the subcapsular/cortex areas. H&E: Hematoxylin and Eosin. LANA1: Latency-associated nuclear antigen-1.

A 76-year-old male with two weeks of fever up to 102.2 °F refractory to oral antibiotics presented to the hospital. On admission, his hemoglobin was 12.5 g/dL, platelets were 93 × 10^9^/L, and white blood cell count was 7.28 × 10^9^/L. His CT scans showed mildly enlarged supraclavicular, axillary, mediastinal, retroperitoneal, and inguinal lymph nodes without hepatosplenomegaly. He was negative for human immunodeficiency virus (HIV), hepatitis B and C, coccidiomycosis, histoplasmosis, quantiferon, and extensive rheumatologic testing. He had elevated C-reactive protein (8.4 mg/dL), erythrocyte sedimentation rate (34 mm/h), and ferritin (4271 ng/mL), an elevated IgG4 of 133 mg/dL and a normal total IgG of 1230 mg/dL (IgG4/IgG ratio of 10.81%). An excisional lymph node biopsy of a right inguinal lymph node revealed NSHL (Figure 1A). HHV8 LANA testing was performed, which was negative (Figure 1B).

An elevated IgG4 level in the setting of fever and elevated inflammatory markers prompted suspicion of Castleman Disease; consequently, HHV8 DNA polymerase chain reaction (PCR) was ordered, with a result of 453,001 copies/mL. IL-6 was elevated at 12.8 pg/mL (reference ≤1.8 pg/mL), but re-review of the lymph node showed only NSHL. For the treatment of stage IIB NSHL, the patient received five cycles of A + AVD as per Echelon-1 [2]. The patient’s fevers rapidly resolved after initiating treatment, and an interim PETCT showed all lymph nodes to be smaller, with reduced 18F-fluorodeoxyglucose (FDG) activity in multiple lymph nodes, consistent with a partial response (Deauville 4).

After five cycles of treatment, the patient remained afebrile but had significant fatigue. A positron emission tomography–computed tomography (PET-CT) scan revealed diffusely increased FDG activity in multiple nodes (Deauville 5). Labs revealed ongoing thrombocytopenia (31 × 10^9^/L), elevated IgG4 (150 mg/dL, total IgG of 1410 mg/dL), IL-6 (1.9 pg/mL), and HHV8 DNA PCR at 154,114 copies/mL. A bone marrow biopsy showed mild hypocellularity (30%) with active trilineage hematopoiesis, normal karyotype, normal flow cytometry, and normal myeloid next-generation sequencing. A core needle biopsy of a cervical lymph node revealed HHV8+ subcapsular germinal center cells, a lymphoid follicle with onion-skin-appearing mantle zones with penetrating hyaline vessel, and 20% IgG4 plasma cells, consistent with HHV8+ Castleman Disease (Figure 1C,D). The patient was then treated with four cycles of rituximab for HHV8-associated MCD with a complete response on PET-CT (Deauville 1), normalization of IgG4, cytopenias, and IL-6 levels, and significant improvement in the patient’s energy level.

## 3. Materials and Methods

**Figure 2:**
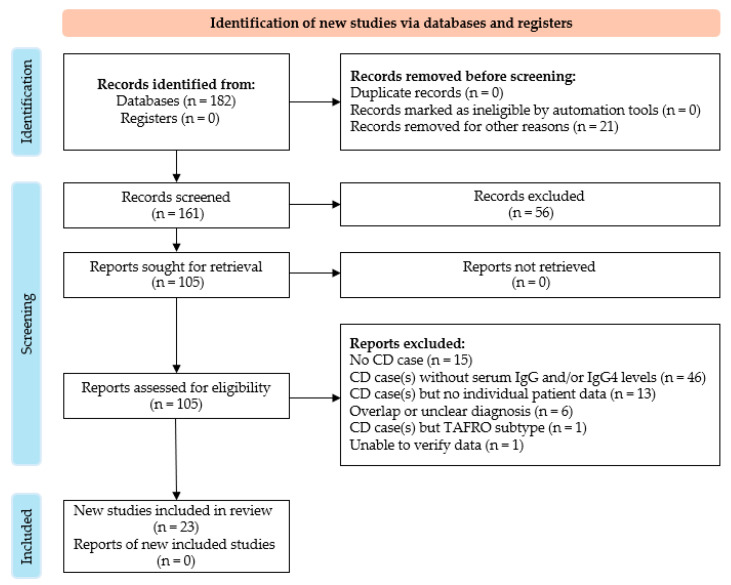
PRISMA flow diagram [3].

For the literature review of the association of CD and IgG4 levels, a Medline search from 2000 to 14 February 2025 was made using the following key words: Castleman AND IgG4. The database search was conducted by 3 authors (A.H., P.K., and E.L.). Only English-language papers were included. All publications reporting 1 or more cases of MCD with individual-patient-level serum IgG and IgG4 levels at the time of diagnosis were included. Exclusion criteria included articles with aggregate data, unclear or overlapping diagnoses, data that could not be verified, and the absence of individual IgG and IgG4 levels. Studies were assessed and excluded by first reading the title and abstract, and then by reading the full text for the studies that required further investigation. Disagreements were discussed, and consensus was reached for each case. Three authors (A.H., P.K., and E.L.) independently extracted data from all studies into data summary tables. This case was presented in accordance with the CARE reporting checklist as can be seen in Figure 2 (Supplementary File).

## 4. Results

A total of 182 publications were searched, of which none were duplicates (Figure 1). These 182 unique articles were screened for eligibility. In total, 21 were excluded for not being written in English and 56 were excluded after reading the title and abstract. Reports were sought for retrieval for the remaining 105 publications. All reports were retrieved. A total of 15 were excluded after reading the full report because there were no cases of MCD, 46 because the MCD cases in the studies lacked serum IgG and/or IgG4 levels, 13 because the data was aggregated, 6 because patients either had overlapping or unclear diagnoses, 1 because it had a TAFRO subtype, and 1 because data was unable to be verified. In total, 23 met full inclusion criteria, and 36 individual cases with individual serum IgG4 and IgG levels were found from the 23 studies. Several of the referenced sources did not provide exact IgG4/IgG plasma cell ratios. As a result, calculating an average would have required excluding those cases, potentially introducing bias and reducing the completeness of our analysis.

## 5. Discussion

MCD is a rare lymphoproliferative disorder that is often difficult to diagnose, both due to a range of nonspecific overlapping signs and symptoms and a lack of formal diagnostic criteria. The inflammatory response in MCD is known to elevate serum IgG4 levels, although this process is not unique to MCD and can be seen in many other disease processes, including rheumatologic conditions that may present similarly with widespread lymphadenopathy, such as IgG4-related disease (IgG4-RD), sarcoidosis, or even solid malignancies such as cholangiocarcinoma, colorectal cancer, lung cancer, or pancreatic cancer [4]. Hodgkin’s lymphoma (HL) can also elevate levels of IgG4 itself, but in our case the patient continued to have a modestly elevated IgG4/IgG ratio after appropriate treatment of HL, which was part of what led to the suspicion of and eventual diagnosis of HHV8-associated iMCD [5]. IgG4-RD often mimics MCD due to their multiple overlapping manifestations, including lymphadenopathy, elevated serum IgG levels, elevated serum IgG4 levels, and the presence of IgG4+ plasma cells in tissues. Because management varies greatly between these disease processes, it is critical to identify distinguishing characteristics. Although the serum IgG4 levels may be elevated to similar degrees in both diseases, there are some notable distinctions [1]. For example, fever is rarely observed in patients with IgG4-RD. In contrast, the pathogenesis of MCD involves overproduction of interleukin-6 (IL-6), which can trigger a cytokine storm and lead to inflammatory symptoms, including fever and elevated inflammatory markers such as C-reactive protein (CRP) [1]. On the other hand, involvement of orbits, lacrimal or salivary glands, or pancreas, atopic history, or non-involvement of a lymph node has high specificity for IgG4-RD, especially when combined with low CRP and IgA [6].

Serum IgG4 and IgG may have a role in distinguishing iMCD and IgG4-RD. A retrospective study by Otani et al. demonstrated that both iMCD and IgG4-RD raise serum IgG4 >135 mg/dL, without a statistically significant difference between them. However, the same study also demonstrated that the degree of elevation of IgG4/IgG is greater in IgG4-RD when compared to MCD: 24.1 (8.7–59.0) vs. 8.6 (1.5–23.6), *p* < 0.001 [1]. Similar serum IgG4/IgG ratios were found in a separate retrospective study by Nishikori et al. comparing IgG4-RD to plasmacytic lymphadenopathy-type idiopathic MCD: 28.2 (25.3–40.8) vs. 12.0 (6.4–20.1) *p* < 0.001 [7]. It should be noted that some iMCD cases, including cases of MCD with associated TAFRO syndrome [8], may show no elevation in IgG4, indicating that the absence of elevated serum IgG4 levels is not 100% sensitive in ruling out MCD [9].

In our systematic review, we found that the average serum IgG4/IgG ratio was 14.61% (0.06–37.78) when including all exact data points (Table 1). This falls below the suggested serum IgG4/IgG receiver operator characteristic (ROC)-derived cut-off value of 19.0% found by Nishikori et al. in their comparison of idiopathic Multicentric Castleman Disease and IgG4-RD, further supporting their work [10]. Chen et al. report in their review article on IgG4-RD that the serum IgG4/IgG ratio is typically >0.2 in patients with IgG4-RD [11]. In our case, the patient’s modestly elevated IgG4/total IgG ratio (9.43%) in the clinical context of elevated inflammatory markers and widespread lymphadenopathy led to the suspicion of MCD. We suggest that IgG4/IgG ratio may be a helpful tool when used in combination with other clinical factors when working up a fever of unknown origin.

Limitations of our review include the small sample size and the lack of inclusion of other causes of IgG4 elevation. Histologic subtypes of Castleman Disease were not reported in each manuscript. Additionally, this review does not include any bulk studies with averaged data as we were unable to confirm that these averages were of all unique cases compared to our other sources, given that many included studies from the same universities, such as Okayama University.

## Figures and Tables

**Table 1:** Summary of case reports and series of IgG4 elevation in Castleman Disease (*n* = 24).

Reference/Year	Age/Sex	Diagnosis	Serum IgG (mg/dL)	Serum IgG4 (mg/dL)	Serum IgG4/IgG (Ratio %)	Plasma Cell Pathology Source	IgG4/IgG Plasma Cell Ratio (Histology)	Fever	IL-6 (pg/mL)
Li et al., 2025 [12]	36/M	iMCD HHV-8-negative	49,900	9869.6	19.78%	LN	~40%	No	179.6
Otoshi et al., 2024 [13]	33/M	All iMCD HHV-8-negative	4341	57	1.31%	All lung	Reported <40%	NR	16
	48/F		5974	72	1.21%				14
	57/F		3846	142	3.69%				35
	40/M		8184	1812	22.14%				20
Suzuki et al., 2024 [14]	47/M	iMCD HHV-8-negative	5660	2113	37.33%	LN	>40%	No	11.6
Sawada et al., 2024 [15]	55/M	iMCD HHV-8-negative	7132	2130	29.87%	LN	~50–60%	Yes	21.1
Takiguchi et al., 2023 [16]	60s/F	iMCD HHV-8-negative	5952	710	11.93%	LN	10–20%	NR	15.6
Kawanishi et al., 2023 [17]	82/F	iMCD HHV-8-negative	8356	1570	18.80%	Kidney	NR	No	12.2
Cheng et al., 2022 [18]	73/M	iMCD HHV-8-negative	5760	1460	25.00%	LN	>40%	No	18.39
Shionoya et al., 2021 [19]	40s/M	iMCD HHV-8-negative	4124	278	6.74%	LN	NR	NR	NR
Nakai et al., 2021 [20]	41/M	iMCD HHV-8-negative	3945	1340	33.97%	LN	>40%	No	11.5
Zhao et al., 2021 [21]	52/M	iMCD HHV-8-negative	5260	1030	19.58%	LN	<10%	No	30.62
Endo et al., 2021 [22]	59/M	iMCD HHV-8-negative	6058	1130	18.65%	LN	>40%	Yes	9.3
Kawano et al., 2021 [23]	53/F	All iMCD HHV-8-negative	8224	625	7.6%	LN	30%	No	15.9
	67/F		5669	738	13.02%	Renal	25%	No	NR
Bonometti et al., 2020 [24] *	56/M	iMCD HHV-8-negative	2960	601	20.30%	LN	NR	No	NR
Katsumata et al., 2018 [25]	67/F	MCD HHV-8-negative	3916	435	11.11%	LN	12.9%	Yes	35.9
						Lung	24.1%		
						LN	36.4%		
Hu et al., 2018 [26]	47/M	MCD (HHV-8 not specified)	7210	>356	>4.94%	LN	>40% (LN) <40% (tubular vascular walls of LN)	No	NR
Takano et al., 2018 [27]	43/M	MCD (HHV-8 not specified)	3172	162	5.10%	NA	NA	No	NR
Izumi et al., 2017 [28]	50/F	MCD (HHV-8 not specified)	4020	231	0.06%	LN	37.2%	No	9.3
Zoshima et al., 2016 [29]	66/M	All iMCD HHV8-negative	4415	987	22.36%	LN	NA	No	13
	64/F		4807	235	4.89%	LN	NA	Yes	113
Ogoshi et al., 2013 [30]	42/F	iMCD HHV-8-negative	2295	867	37.78%	LN	50.5%/56.0%	Yes	19.9
	50/F		4831	1160	24.01%		61.5%/51.5%	No	13.6
	58/M		4080	389	9.53%		42.5%/46.5%	No	19.7
	43/F		6915	294	4.25%		20.0%/8.0%	No	33.1
Gatti-Mays et al., 2012 [31]	70/M	iMCD HHV-8-negative	3530	39.6	1.12%	LN	NR	NR	NR
Takeuchi et al., 2012 [32]	55/F	iMCD HHV-8-negative	5180	1490	28.76%	Skin	40.6	NR	11.5
Oshitari et al., 2010 [33]	79/M	MCD (HHV8 not specified)	2790	695	24.9%	Lacrimal gland	NR	NR	5.1
Sato Y et al., 2010 [34]	43/M	All iMCD HHV-8-negative	4003	93	2.30%	All LN	45.2%	No	8.6
	68/M		3591	147	4.1%		54.7%	No	19.6
	47/F		3113	154	4.9%		46.5%	No	27.4
	50/M		5082	314	6.2%		48.8%	Yes	31.2
	65/M		8380	1460	17.4%		76.6%	No	30.0
	54/M		7957	789	11.5%		43.4%	No	17.3

* Bonometti et al. report a case of iMCD with many features consistent with POEMS; however, there is no electrophoresis reported.

## Data Availability

All data listed in the article.

## Supplementary Materials

The following supporting information can be downloaded at: https://www.mdpi.com/article/10.3390/hematolrep18020026/s1, Supplementary File: CARE Checklist of information to include when writing a case report.

## Abbreviations

The following abbreviations are used in this manuscript:

CD	Castleman Disease
DL-PCD	Plasma Cell-Type Castleman Disease with Diffuse Parenchymal Lung Involvement
F	Female
HHV8	Human Herpesvirus 8
IgG	Immunoglobulin G
IgG4	Immunoglobulin G4
IL-6	Interleukin-6
iMCD	Idiopathic Multicentric Castleman Disease
LN	Lymph Node
M	Male
MCD	Multicentric Castleman Disease
N	Number
NA	Not Applicable
NR	Not Reported
TAFRO	TAFRO Syndrome
